# Cloning, purification, and characterization of an organic solvent-tolerant chitinase, MtCh509, from *Microbulbifer thermotolerans* DAU221

**DOI:** 10.1186/s13068-018-1299-1

**Published:** 2018-11-08

**Authors:** Hyo-Jung Lee, Yong-Suk Lee, Yong-Lark Choi

**Affiliations:** Department of Biotechnology, Dong-A University, Busan, 49315 Republic of Korea

**Keywords:** *Microbulbifer thermotolerans*, DAU221, Organic solvent-tolerant chitinase, Transglycosylation

## Abstract

**Background:**

The ability to use organic solvents in enzyme reactions offers a number of industrially useful advantages. However, most enzymes are almost completely inactive in the presence of organic solvents. Thus, organic solvent-tolerant enzymes have potential applications in industrial processes.

**Results:**

A chitinase gene from *Microbulbifer thermotolerans* DAU221 (*mtch509*) was cloned and expressed in *Escherichia coli* BL21 (DE3). The molecular weight of the expressed MtCh509 protein was approximately 60 kDa, and it was purified by His-tag affinity chromatography. Enzymatic assays showed that the optimum temperature for MtCh509 chitinase activity was 55 °C, and the enzyme was stable for 2 h at up to 50 °C. The optimum pH for MtCh509 activity was in the sub-acidic range, at pH 4.6 and 5.0. The activity of MtCh509 was maintained in presence of 1 M salt, gradually decreasing at higher concentrations, with residual activity (20%) detected after incubation in 5 M salt. Some organic solvents (benzene, DMSO, hexane, isoamyl alcohol, isopropyl alcohol, and toluene; 10–20%, v/v) increased the reactivity of MtCh509 relative to the aqueous system. When using NAG_3_, as a substrate, MtCh509 produced NAG_2_ as the major product, as well as NAG_4_, demonstrating that MtCh509 has transglycosylation activity. The *K*_m_ and *V*_max_ values for MtCh509 using colloidal chitin as a substrate were 9.275 mg/mL and 20.4 U/mg, respectively. Thus, MtCh509 could be used in extreme industrial conditions.

**Conclusion:**

The results of the hydrolysate analysis and the observed increase in enzyme activity in the presence of organic solvents show that MtCh509 has industrially attractive advantages. This is the first report on an organic solvent-tolerant and transglycosylating chitinase from *Microbulbifer* species.

## Background

Chitin is a linear β-1,4-linked homopolymer of *N*-acetyl-β-d-glucosamine (GlcNAc), the second most abundant biomass on earth after cellulose. Chitin is present in the cell walls of fungi and in the exoskeletons of crustaceans and arthropods. Approximately 10^11^ tons of chitin is discarded every year [1, 2]. However, chitin is biocompatible, biodegradable, and bioabsorbable [3]. Commercially, chitin is traditionally degraded with concentrated acids or alkalis. However, there are many issues with these processes, including the production of environmental pollution and acidic waste, low yield, and high cost. Enzymatic hydrolysis of chitin could resolve these problems. Therefore, the development of an enzymatic method of chitin degradation is important [4–8].

Chitinases [EC. 3.2.1.14] are hydrolytic enzymes that degrade the glycosidic bonds between chitin polymers. These enzymes belong to glycosyl hydrolase family 18 (GH 18) and 19 (GH 19) with most chitinases in bacteria, fungi, viruses, animals, plants, and other organisms being GH18 enzymes [9, 10]. All GH 18 chitinases have a triosephosphate isomerase fold [TIM (*β*/*α*)_8_ fold] with a conserved DXXDXDXE motif in the catalytic domain. The TIM (*β*/*α*)_8_ fold contains *α* + *β* insertion domain that is associated with deepening of the substrate-binding groove [11]. The deepening of the substrate-binding groove influences enzymatic activity, i.e., processivity. In a previous report on processive cellulases, it was shown that processive chitinases have long substrate-binding clefts, or tunnels, as well as substrate-binding clefts [12, 13]. Processive enzymes have increased catalytic efficiency because the detached polymer chains are prevented from reassociating with crystalline material and stay closely associated with polymer chains [12, 14].

Chitinases can be classified as endochitinases or exochitinases. Endochitinases randomly cleave internal sites in the chitin chain and release GlcNAc and chitooligosaccharides, whereas exochitinases or chitobiosidases (EC 3.2.1.29) catalyze the hydrolysis of chitin polymers from the reducing or non-reducing ends and release chitobiose. 1,4-β-*N*-Acetylglucosaminidases [EC 3.2.1.30] cleave chitobiose and release *N*-acetylglucosamine [15–17].

Some chitinases have accessory domains, such as chitin-binding domains (ChBDs), fibronectin type III domains (FN3s), and polycystic kidney disease domains (PKDs). These domains help the chitinase cleave polymeric substrates. In addition, some chitinases have transglycosylation (TG) activity. Through TG, the produced chitooligosaccharide moiety can be transferred to a proper acceptor to make a new glycosidic bond. Furthermore, TG allows chitooligosaccharide with a specific degree of polymerization (DP) to be produced [18]. *N*-Acetylglucosamine and chitooligosaccharides produced via the hydrolase or TG activity of chitinase have useful functions, including as drug delivery carriers, antioxidants, in hemostasis and wound healing, immuno-enhancers and host defense activation, blood cholesterol control, antibacterial agents for food preservation, elicitors, lysozyme inducers, immuno-enhancers, and for natural cancer prevention and treatment [3, 16, 19, 20].

In a previous report, *Microbulbifer thermotolerans* DAU221 was isolated from the eastern coast of the Republic of Korea, and genomic library of this bacterium was constructed [21]. In this study, we report the characterization of novel chitinase from DAU221. The putative chitinase was cloned, expressed, purified, and characterized, and the results demonstrated that it is a sub-acidic chitinase with transglycosylation activity. The results of this study indicate that the chitinase could be used in extreme industrial conditions.

## Materials and methods

### Preparation of colloidal chitin

Colloidal chitin was prepared by a modification of the method of Roberts and Selitrennikoff [22]. Briefly, 175 mL of concentrated HCl was carefully poured onto 10 g of crab shell (C9213; Sigma-Aldrich, St. Louis, USA) and stirred at 4 °C for 24 h. Then, 1 L of ice-cold 95% ethanol was added to the mixture and stirred at 4 °C for 12 h. The sediment was harvested by centrifugation at 8000 rpm for 20 min at 4 °C, and the continually washed with sterile distilled water until the acid was neutralized.

### Bacterial strains, plasmids, and culture conditions

The *Microbulbifer thermotolerans* DAU221 strain used in this study was deposited in the Korean Culture Center of Microorganisms (KCCM 43021; 16S rDNA sequence GenBank ID: KC571186). It was cultivated in Marine Broth 2216 (MB; Difco, Detroit, MI, USA). Plasmids pUC118 and pCC1FOS (Epicentre, Madison, WI, USA) and *Escherichia coli* (*E. coli*) JM109, EPI300-T1 were used to construct the genomic library and cloning the chitinase gene. The pCold I plasmid vector (TaKaRa, Otsu, Japan) and *E. coli* BL21 (DE3) cells were used for heterologous expression. *M. thermotolerans* DAU221 was cultured in MB medium with shaking at 30 °C overnight. *E. coli* JM109 and BL21 (DE3) were grown in Luria–Bertani (LB) broth at 37 °C. Ampicillin (50 μg/mL) or chloramphenicol (12.5 μg/mL) was added to the LB broth when required. Oligonucleotide primers were purchased from Bioneer (Daejeon, South Korea). Chitooligosaccharides—*N*-acetylglucosamine (NAG_1_), chitobiose (NAG_2_), chitotriose (NAG_3_), chitotetraose (NAG_4_), chitopentaose (NAG_5_), and chitohexaose (NAG_6_)—were purchased from Seikagaku (Tokyo, Japan).

### Cloning and amino acid sequence analysis of the chitinase from *M. thermotolerans* DAU221

*Microbulbifer thermotolerans* DAU221 genomic library was constructed. The library was constructed using a fosmid library construction kit (CopyControl Fosmid Library Production Kit; Epicentre) [21]. The genomic library was cultured on LB agar plates containing 0.2% (w/v) colloidal chitin and ampicillin (50 μg/mL) at 37 °C for 5 days. After incubation, colonies with clear halo zones were chosen as chitinase-producing clones. To make the first subclone, *Hin*dIII was used to cut out *mtch509*, which was ligated into pUC118. Then, the chitinase-producing clone was digested with *Xba*I and ligated into pUC118 to make a second subclone. Both of these had chitinase activity, and the sequences were confirmed.

Sequences that are similar to MtCh509 were searched by BLAST (National Center for Biotechnology Information, NCBI). The presence of a signal peptide was investigated using the SignalP 3.0 server (http://www.cbs.dtu.dk/services/SignalP) [23]. MtCh509 was aligned with similar sequences using the ClustalW [24] and ESPript 3.0 programs [25]. Three-dimensional (3D) structure of MtCh509 was predicted with Protein Homology/analogY Recognition Engine Ver. 2.0 (Phyre^2^) server (http://www.sbg.bio.ic.ac.uk/phyre/) [26].

### Expression and purification of recombinant MtCh509

To express recombinant MtCh509, *E. coli* BL21 (DE3) cells containing a recombinant plasmid were incubated in LB broth containing 50 μg/mL ampicillin at 37 °C. When the OD_600_ reached 0.4–0.5, the cells were incubated on ice for 30 min. Then, IPTG was added to a final concentration of 0.1 M, and incubated at 15 °C for 24 h. The cells were harvested by centrifugation at 6000 rpm for 20 min at 4 °C. The cells were re-suspended with His-tag binding buffer [20 mM sodium phosphate (pH 7.4), 0.5 M NaCl, 5 mM imidazole]. The cells were lysed by an ultrasonicator with 15-s pulse for three times. The lysed cells were centrifuged (13,000 rpm at 4 °C for 10 min), and then the supernatant was collected. The recombinant protein was purified with His-Trap HP column (Amersham Biosciences). The column was equilibrated with His-tag binding buffer, and the collected supernatant was loaded on the column. The bound protein was eluted with His-tag elution buffer [20 mM sodium phosphate (pH 7.4), 0.5 M NaCl, 0.5 M imidazole]. The eluted fractions were passed through an Amicon Ultra-4 filter (Millipore, Bedford, MA, USA), and the buffer was changed to 20 mM sodium phosphate (pH 7.4).

### Determination of protein quantification

Purified protein was quantified by the Bradford method [27]. Bovine serum albumin (BSA) was used to construct a standard calibration curve.

### Determination of molecular weight and zymogram analysis

The molecular weight of MtCh509 was determined using sodium dodecyl sulfate–polyacrylamide gel electrophoresis (SDS-PAGE) method [28]. The SDS-PAGE gel consisted of a 10% separating gel and a 5% stacking gel. The protein size was estimated using standard protein markers (Elpis-Biotech, Daejeon, Korea). After separating the samples, the gel was stained with 0.05% Coomassie brilliant blue R-250 for 2 h, and then decolorized with de-staining solution (water:methanol:acetic acid glacial = 6:3:1). To detect chitinase activity, the protein sample was diluted in native page buffer and loaded into a gel containing 0.1% glycol chitin. After electrophoresis, the gel was incubated in refolding buffer [50 mM citrate buffer (pH 5.0), 1% Triton X-100] at 50 °C for 4 h. Then, the gel was stained with 0.01% calcofluor white M2R in 50 mM citrate buffer (pH 5.0) for 2 h. The gel was rinsed several times with distilled water for 1 h each and visualized on a UV transilluminator [29].

### Chitinase activity

Chitinase activity was measured using a modified dinitrosalicylic acid (DNS) method [30]. The reaction mixture (total volume, 250 μL) contained 50 mM citrate buffer (pH 5.0), 1% colloidal chitin (100 μL), and 3 μg of purified enzyme. The reaction mixture was incubated at 55 °C for 1 h. After the reaction was complete, DNS (750 μL) was added to the reaction mixture and boiled for 10 min. Then, the mixture was cooled on ice and centrifuged at 13,000 rpm for 1 min. Reducing sugar was measured as the absorbance at 540 nm. One unit of MtCh509 chitinase activity was defined as the amount of enzyme needed to liberate 1 μmol of reducing sugar per minute. NAG_2_ was used as a standard.

### Effect of temperature and pH on chitinase activity

To determine the optimum temperature for MtCh509 chitinase activity, chitinase assays were performed at various temperatures (10–80 °C). All other reaction parameters were standard assay conditions. Thermal stability was measured as the residual activity after purified MtCh509 was preincubated at 10–80 °C for 30 min before the assay. For the assay, 1% colloidal chitin was added to the preincubated sample, and the reaction mixture was incubated at the optimum temperature for 1 h. To assess high-temperature stability, the enzyme was preincubated at 40, 50, 55, and 60 °C for up to 4 h and then incubated at the optimum temperature for 1 h.

The optimum pH was determined by performing the assay with a variety of buffers at different pH values (50 mM each): citrate buffer (pH 3.0–6.0), sodium phosphate (pH 6.0–8.0), Tris–HCl (pH 8.0–9.0), and glycine–NaOH (pH 9.0–10.0) at the optimum temperature for 1 h. To determine pH stability, purified MtCh509 was preincubated in the buffers mentioned above for 1 h on ice. After preincubation, 1% colloidal chitin (100 μL) was added to the mixture, which was incubated at the optimum temperature for 1 h.

### Effects of metal ions, chemicals, NaCl, and organic solvents on chitinase activity

The effect of metal ions and NaCl was assayed by measuring the residual activity in the presence of each ion or salt. Purified MtCh509 was preincubated with 1, 5, and 10 mM of various metal ions, including Ba^2+^, Ca^2+^, Co^2+^, Cs^2+^, Cu^2+^, Fe^3+^, Hg^2+^, K^+^, Li^+^, Mg^2+^, Na^+^, Ni^+^, and Zn^2+^, as well as EDTA and dithiothreitol [DTT] for 1 h on ice. Then, to assess residual activity, 1% colloidal chitin was added to the mixture and incubated for 1 h at the optimum temperature and pH. Because EDTA is a metal ion chelator, it was used as a negative control [16]. To determine the effect of salinity on activity, the enzyme was incubated with different concentrations of NaCl (0.5, 1, 1.5, 2, 2.5, 3, 4, and 5 M). Purified MtCh509 mixed with NaCl was placed on ice for 30 min. Then, 1% colloidal chitin was added to the preincubated mixture and incubated under standard assay conditions. The effect of organic solvents on chitinase activity was studied by incubating the enzyme with different solvents, including acetone, butanol, DMSO, ethanol, hexane, isoamyl alcohol, isopropyl alcohol, and methanol. The final concentrations of organic solvents were 10% and 20% (v/v). The reaction mixtures, containing 50 mM citrate buffer (pH 5.0), 1% colloidal chitin, organic solvent, and purified MtCh509, were incubated at 55 °C for 1 h. Then, residual activity was measured by the DNS methods.

### Substrate specificity and enzyme kinetics of MtCh509

To determine the substrate specificity of MtCh509, purified MtCh509 was incubated with a variety of substrates under standard assay conditions. The final concentration of each substrate was 1%. The substrates tested were colloidal chitin, glycol chitin, carboxymethyl cellulose (CMC), and Avicel (microcrystalline cellulose). The reducing sugar released in each reaction was measured using the DNS method as mentioned above. The kinetic parameters of MtCh509 chitinase activity were estimated by studying the initial reaction rate of purified MtCh509. Different concentrations of colloidal chitin were used as substrates at final concentration ranges of 2–7 mg/mL. The reaction mixtures were incubated in assay buffer for 10 min. The assay was performed in triplicate. The kinetic constants, *K*_m_ and *V*_max_, were estimated by a Lineweaver–Burk plot.

### Thin-layer chromatography (TLC) analysis of colloidal chitin and chitooligosaccharides hydrolysates produced by MtCh509

Reaction mixtures (25 μL) containing 1% colloidal chitin (10 μL) and 0.3 μL of purified enzyme in 50 mM citrate buffer (pH 5.0) were incubated at 55 °C for various times (0, 1, 15, 30, 60, 180, 360, 540, and 720 min). When chitooligosaccharides (NAG_2_–NAG_6_) were used as substrates, the reaction mixtures (10 μL; 50 mM citrate buffer, substrate, and purified MtCh509) were incubated at 55 °C for 1, 5, 10, 15, 30, and 60 min. Then, 1 μL of 0.1 M NaOH was added to stop the reaction. When the reaction was complete, aliquots of the reaction mixtures were separated by TLC on a silica gel plate (Dieselgel 60; Merck, Berlin, Germany) with a solvent system containing *n*-butanol, methanol, 25% ammonia solution, and water [5:4:2:1 (v:v:v:v)]. The products were detected by spraying the plate with aniline-diphenylamine reagent (4 mL of aniline, 4 g of diphenylamine, 200 mL of acetone, and 30 mL of 85% phosphoric acid) and baking it at 180 °C for 10 min [31].

### High-performance liquid chromatography (HPLC)

To identify the major products of chitin hydrolysis by MtCh509, an HPLC analysis was performed. Colloidal chitin was used as substrate. Reaction mixtures (500 μL) containing colloidal chitin, 50 mM citrate buffer (pH 5.0), and 6 μg of MtCh509 were incubated at 55 °C for 12 h. After incubation, the reaction mixture was centrifuged at 13,000 rpm for 10 min at 4 °C. Then, the supernatant was filtered through a 0.22-μm syringe filter. The filtered reaction mixture was injected into HPLC (Waters 1500 series HPLC system) equipped with an Inertsil an HPLC column (4.6 × 250 mm, 5 μm; GL Sciences Inc., Japan). The injection volume was 20 μL. The column temperature was 40 °C. The mobile phase was composed of 70% acetonitrile and 30% MilliQ H_2_O, and the flow rate was 1 mL/min. ELSD was used as the detector and the carrier gas was nitrogen, which was delivered at 20 psi. The drift temperature was 50 °C. An equal quantity of chitooligosaccharides (NAG_1_–NAG_6_) was used to generate standard peaks.

## Results

### Isolation and amino acid analysis of MtCh509

**Fig. 1:**
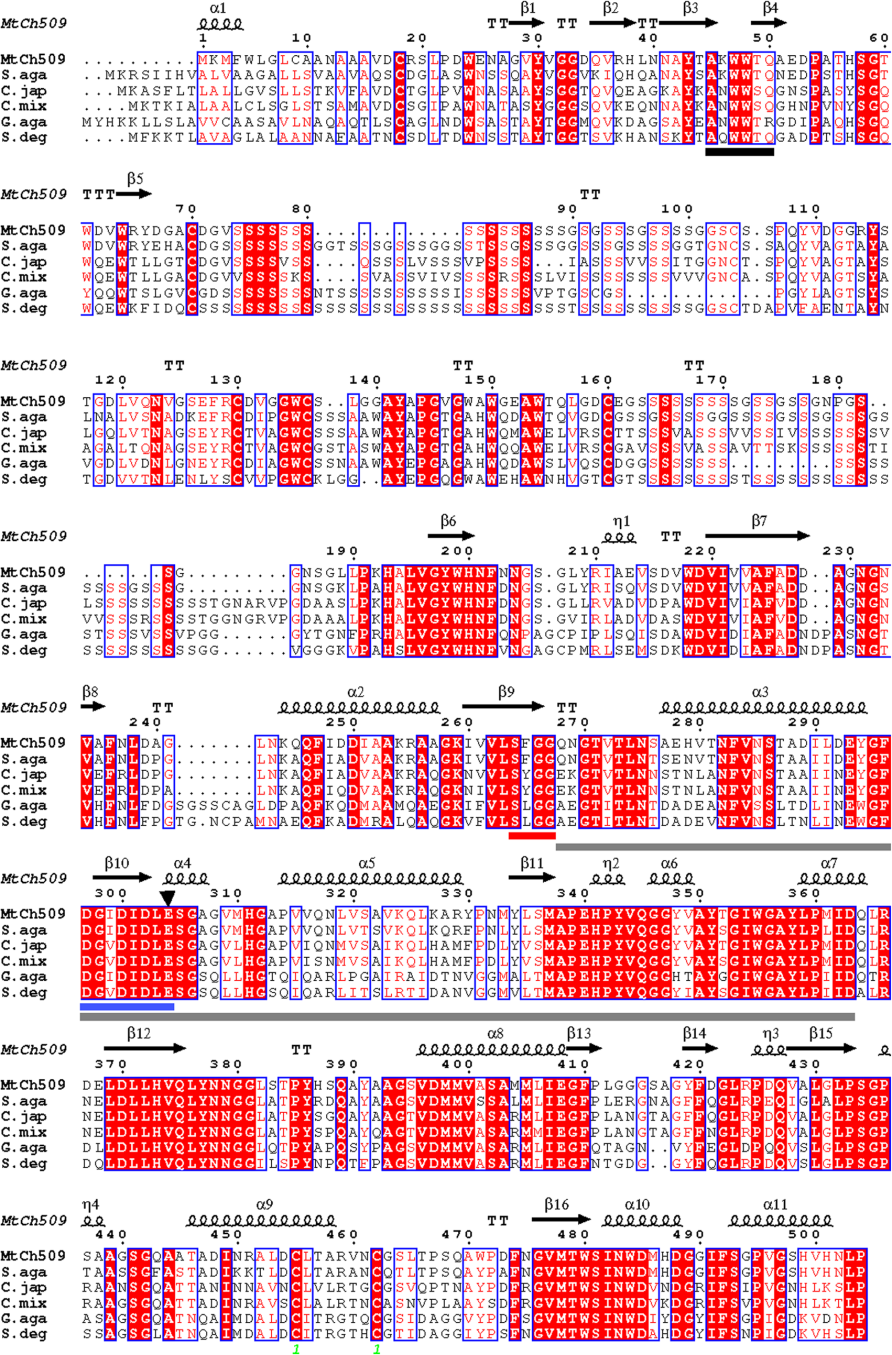
Alignment of the MtCh509 amino acid sequence with other bacterial chitinases. Similar sequences are marked by boxes and identical sequences are highlighted in red. The chitin-binding domain and the SXGG and DXXDXDXE motifs are marked with black, red, and blue lines under the sequences, respectively. The conserved catalytic proton donor is marked with a black inverted triangle. The conserved *α* + *β* insertion domain is marked with a gray line. Secondary structural elements (i.e., alpha helix [*α*], beta sheet [*β*], random coil [*ƞ*], and beta turn [*T*]) are marked on the MtCh509 sequence. MtCh509: chitinase from *M. thermotolerance* DAU221 (WP_06714446); S. aga: glycosyl hydrolase from *Simiduia agarivorans* (WP_015046629); C. jap: glycosyl hydrolase from *Cellvibrio japonicus* (WP_012488573); C. mix: glycosyl hydrolase from *Cellvibrio mixtus* (WP_039915554); G. aga: glycosyl hydrolase from *Gilvimarinus agarilyticus* (WP_041522559); and S. deg: glycosyl hydrolase from *Saccharophagus degradans* (WP_011468184)

Chitin degrading clones identified among the fosmid genomic library of *M. thermotolerans* DAU221 were isolated on LB-colloidal chitin agar medium and named CH1-CH4. The CH4 clone was selected and subcloned into pUC118 for sequencing, resulting in the identification of the chitinase gene, *mtch509*, from *M. thermotolerans* DAU221. The *mtch509* gene is 1527 bp and encodes a protein of 509 amino acids. The deduced amino acid sequence of MtCh509 was compared with those of known bacterial chitinases, which were obtained from the NCBI database. The protein with the highest amino acid sequence identity (71%) was a chitinase from *Simiduia agarivorans* (WP_015046629). The next highest sequence similarity (63%) was to a chitinase from *Cellvibrio japonicus* (WP_012488573), followed by chitinases from *Cellvibrio mixtus* (WP_039915554, 60%), *Gilvimarinus agarilyticus* (WP_041522559, 56%), and *Saccharophagus degradans* (WP_011468184, 55%). The MtCh509 amino acid sequence contains a glycosyl hydrolase family 18 (GH 18) and chitin-binding domain type 3 (ChtBD3) motifs. The conserved amino acid sequences in the catalytic domain of GH 18 proteins are DXXDXDXE and SXGG, and these sequences are present in MtCh509. In addition, a ChtBD3 motif was also observed to be conserved in MtCh509 (AKWWTQ; Fig. 1).

### Expression and purification of MtCh509

**Fig. 2:**
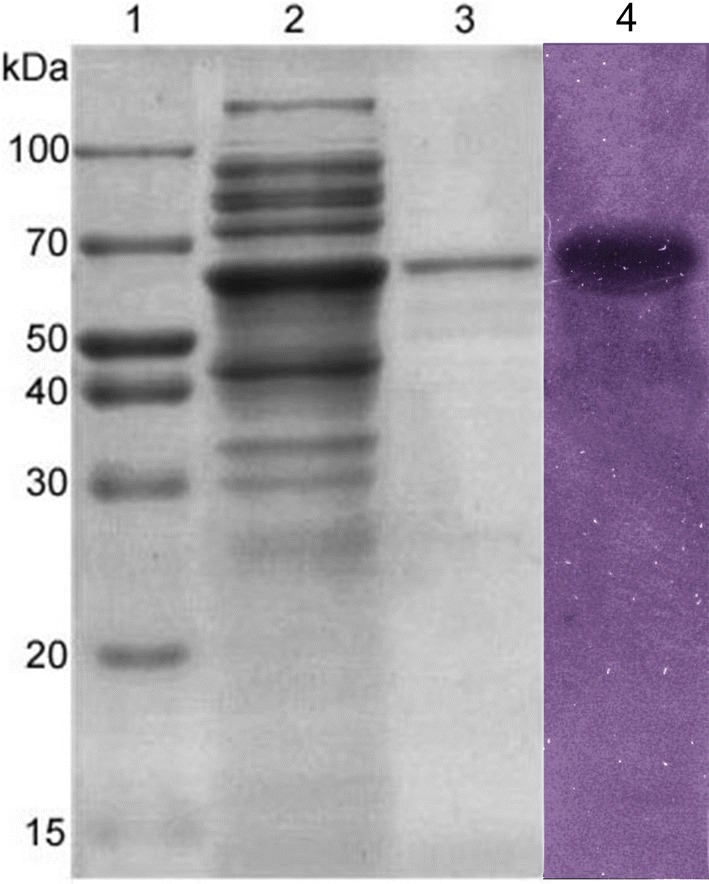
SDS-PAGE analysis of MtCh509 from *M. thermotolerans* DAU221. Lane 1, Protein molecular mass marker; lane 2, soluble protein extracted from *E. coli* cells over-expressing His_6_-MtCh509; lane 3, MtCh509 purified by His-tag affinity chromatography; lane 4, zymogram of MtCh509

The *mtch509* gene was PCR amplified without its signal peptide. The amplicon was cloned into the vector pCold I, such that the resulting plasmid, pCold I-MtCh509, encoded MtCh509 with a 6 histidine tag at the N-terminus. The pCold I-MtCh509 plasmid was transformed in *E. coli* BL21 (DE3) cells for heterologous protein expression. Cells were incubated with 0.1 M IPTG at 15 °C for 24 h after which the cells were disrupted, and the cell lysate containing MtCh509 was collected. The enzyme was purified by His-tag affinity chromatography. An SDS-PAGE analysis under denaturing conditions showed that the molecular weight of MtCh509 was approximately 60 kDa. The zymogram results showed a clear zone around a single band (Fig. 2).

### Effects of temperature and pH on MtCh509 activity

**Fig. 3:**
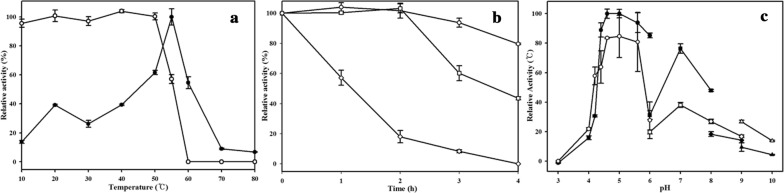
Effect of temperature, pH, and NaCl on MtCh509. **a** Optimal temperature (solid circles) and thermostability (open circles) of MtCh509 under preincubation for 30 min. **b** Further thermostability of MtCh509 preincubated at 40 °C (open circles), 50 °C (open squares), and 55 °C (open diamonds) for 1–4 h. **c** Optimal pH (solid) and pH stability (open) of MtCh509. Reactions were performed in the following 50 mM buffers: citrate buffer for pH 3.0–6.0 (circles), sodium phosphate buffer for pH 6.0–8.0 (squares), Tris–HCl buffer for pH 8.0–9.0 (diamonds), and glycine–NaOH buffer for pH 9.0–10.0 (triangles). The means of the relative values (*n *= 3) and standard deviations are shown

To determine the optimum temperature for the chitinase activity of purified MtCh509, the enzyme was incubated at various temperatures (10–80 °C) for 1 h. Purified MtCh509 showed the highest activity at 55 °C. When the maximal activity was set to 100%, MtCh509 showed ~ 60% activity at 50 °C and 55% activity at 60 °C. When purified MtCh509 was preincubated at 70 °C, the activity decreased sharply to 9% (Fig. 3a). Next, purified MtCh509 was incubated without substrate for 4 h at 40, 50, and 55 °C, and then assayed for chitinase assay. The results showed that the purified MtCh509 was highly stable for 4 h at up to 40 °C. In addition, the enzyme was stable at 50 °C for 2 h (Fig. 3b).

The effect of pH on MtCh509 activity was assessed across a broad range of pH values (3.0–10.0) using 1% colloidal chitin as a substrate (Fig. 3c). The maximal MtCh509 activity was observed in 50 mM citrate buffer at pH 4.6 and 5.0. This activity was significantly increased at pH 4.4 with more than 80% activity observed in 50 mM citrate buffer at pH 4.4–6.0. This activity of MtCh509 decreased sharply (to ~ 30%) in 50 mM sodium phosphate buffer at pH 6.0, increasing to ~ 80% at pH 7.0. Under alkaline conditions, the activity of MtCh509 decreased. MtCh509 exhibited more than 60% stability in 50 mM citrate buffer in the sub-acidic range of pH 4.2–6.0. However, the enzyme exhibited relatively low stability under neutral and alkaline conditions.

### Effects of various metals ion, chemical reagents, and NaCl on MtCh509 activity

To examine the effects of various metal ions and chemical reagents on MtCh509 activity, the purified recombinant enzyme was preincubated with various metal ions and chemical reagents, including Ni^2+^, Cu^2+^, Mg^2+^, Li^+^, Ba^2+^, K^+^, Zn^2+^, Mn^2+^, Ca^2+^, Co^2+^, Cs^2+^, Hg^2+^, EDTA, and DTT, for 1 h on ice without substrate. The final concentrations of metal ions and chemical reagents were 1, 5, or 10 mM. Subsequently, the residual activity of MtCh509 was estimated using colloidal chitin as a substrate at 55 °C for 1 h. In the presence of 1 mM Ni^2+^, MtCh509 showed ~ 100% residual activity decreasing stepwise, in the presence of 5 and 10 mM Ni^2+^. Relatively high activity was maintained in the presence of 5 mM Cu^2+^, although the activity dropped sharply in the presence of 10 mM Cu^2+^. When preincubated with Mg^2+^, the residual activity of MtCh509 was high in the presence of 1 mM Mg^2+^ and decreased moderately in 5 and 10 mM. MtCh509 activity was maintained at relatively high levels in the presence of 5 mM Li^+^, Ba^2+^, and K^+^ but was moderately inhibited in the presence of 10 mM of these metal ions. Residual activity was relatively high and moderate in the presence of 5 mM Zn^2+^ and Co^2+^, respectively, but sharply decreased in the presence of 10 mM of these metals. MtCh509 activity was strongly inhibited at both high and low concentrations of Cs^2+^, and Hg^2+^ strongly decreased MtCh509 activity, even at low concentrations. MtCh509 activity was moderately inhibited by 10 mM Ca^2+^, Mn^2+^, and DTT, and EDTA insignificantly inhibited activity at all assayed concentrations (Table 1).

**Table 1:** Effect of different metal ions and chemical reagents on MtCh509

Substances	Relative activity (%)		
	1 mM	5 mM	10 mM
Control	100	100	100
Ni^2+^	100 ± 5.8	69 ± 6.9	32 ± 1.8
Cu^2+^	99 ± 5.6	91 ± 4.9	6 ± 1.8
Mg^2+^	93 ± 5.2	67 ± 6.9	66 ± 3.9
Li^+^	86 ± 6.3	85 ± 1.6	67 ± 7.4
Ba^2+^	81 ± 1.0	82 ± 0.4	73 ± 1.3
K^+^	82 ± 2.8	84 ± 0.9	50 ± 3.4
Zn^2+^	87 ± 0.8	77 ± 0.0	20 ± 0.6
Mn^2+^	66 ± 3.2	49 ± 0.9	50 ± 1.1
Ca^2+^	59 ± 2.5	51 ± 1.6	51 ± 6.0
Co^2+^	58 ± 2.6	52 ± 1.6	25 ± 2.0
Cs^2+^	40 ± 2.0	37 ± 5.3	48 ± 3.1
Hg^2+^	7 ± 0.5	N.D.	N.D.
EDTA	92 ± 7.3	90 ± 5.0	82 ± 2.2
DTT	52 ± 3.5	59 ± 1.8	67 ± 2.3

±, standard error; ND, not detected

**Fig. 4:**
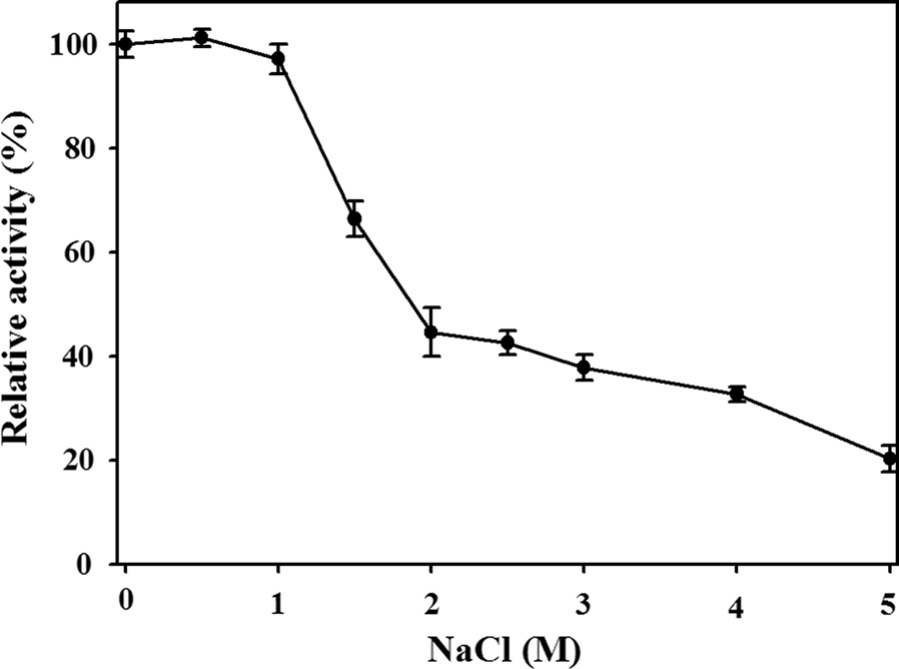
Effect of NaCl on MtCh509. MtCh509 was preincubated on ice for 30 min with various concentrations of NaCl (0.5–5 M). The negative control was a reaction performed in 50 mM citrate buffer (pH 5.0) at 55 °C without NaCl. The means of the relative values (*n *= 3) and standard deviations are shown

The effect of NaCl on MtCh509 was assessed in 50 mM citrate buffer (pH 5.0). Briefly, purified MtCh509 was preincubated with various concentrations of NaCl (0.5–5 M) on ice. Next, residual MtCh509 activity was measured at 55 °C for 1 h and was compared to the activity measured under standard assay conditions without a NaCl preincubation. Approximately 100% enzyme activity was retained after a preincubation with up to 1 M NaCl, and MtCh509 activity decreased to ~ 65% and ~ 45% in the presence of 2 M NaCl. The activity of MtCh509 decreased as the concentration of NaCl increased, and ~ 20% activity was observed in the presence of 5 M NaCl (Fig. 4).

### Effects of various organic solvents on MtCh509 activity

The effects of solvents on MtCh509 chitinase activity were examined by adding various organic solvents (acetone, butanol, DMSO, ethanol, isoamyl alcohol, isopropyl alcohol, methanol, hexane, benzene, toluene, and acetonitrile) to the standard assay mixture, which was incubated for 1 h at 55 °C. The activity of MtCh509 was strongly inhibited by acetone, ethanol, and isopropyl alcohol. In the presence of butanol, MtCh509 activity was moderately inhibited, and when 10% methanol was added, MtCh509 activity was relatively high. In addition, MtCh509 activity rapidly decreased when a high concentration of methanol (20%) was added. In contrast, MtCh509 activity increased in the presence of DMSO, isoamyl alcohol, hexane, benzene, and toluene to > 100%, and activity reached ~ 200% in the presence of 20% benzene (Table 2).

**Table 2:** Effect of various organic solvents on MtCh509 from *M. thermotolerans* DAU221

Solvent	Log *P*_ow_	Relative activity (%)	
		10% (v/v)	20% (v/v)
Control	–	100	100
DMSO	− 1.35	139 ± 0.4	158 ± 9.2
Methanol	− 0.76	84 ± 2.7	9 ± 1.1
Acetone	− 0.24	31 ± 3.0	9 ± 0.5
Ethanol	− 0.24	44 ± 2.1	19 ± 0.8
Acetonitrile	− 0.34	20 ± 0.6	6 ± 0.2
Isopropyl alcohol	0.16	33 ± 0.0	16 ± 0.9
Butanol	0.80	74 ± 3.2	77 ± 8.0
Isoamyl alcohol	1.28	127 ± 4.1	103 ± 6.4
Benzene	2.13	148 ± 29.0	201 ± 7.1
Toluene	2.40	165 ± 5.0	157 ± 17.5
Hexane	3.50	139 ± 12.5	173 ± 29.5

±, standard error

### Substrate specificity and kinetic parameters of MtCh509

Purified MtCh509 activity was assayed using different substrates with the highest activity observed when glycol chitin was used as a substrate. When colloidal chitin was used as a substrate, MtCh509 activity was slightly lower than that observed with glycol chitin. In the presence of CMC, only weak MtCh509 activity was detected, and no activity was detected with Avicel (Table 3). The kinetic parameters of MtCh509 activity, including *K*_m_ and *V*_max_, were determined using a Lineweaver–Burk Plot. The calculated *K*_m_ and *V*_max_ values were 9.275 mg/mL and 20.4 U/mg, respectively (data not shown).

**Table 3:** Substrate specificities of the purified MtCh509 from *M. thermotolerans* DAU221

Substrate	Specific activity (U/mg)	Relative activity (%)
Colloidal chitin	34.256	100
Glycol chitin	40.233	117.4 ± 1.5
Carboxymethyl cellulose	0.503	1.46 ± 1.4
Avicel	ND	ND

±, standard error; ND, not detected

### TLC analysis of the hydrolysates from colloidal chitin and chitooligosaccharides produced by MtCh509

**Fig. 5:**
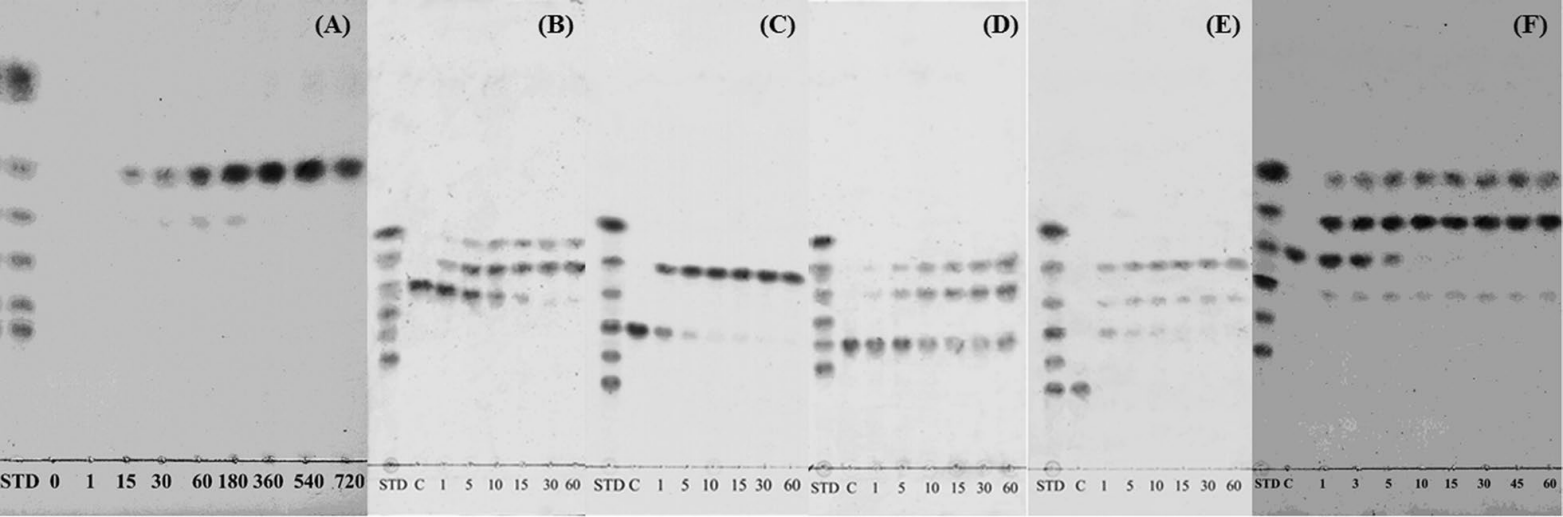
TLC analysis of the hydrolysis products of colloidal chitin and chitooligosaccharide (NAG_3_–NAG_6_) generated by purified MtCh509. The reaction mixture (25 μL), which contained 0.3 μg of purified MtCh509, 50 mM citrate buffer (pH 5.0), and substrates, was incubated at 55 °C for various times (0, 1, 15, 30, 60, 180, 360, 540, or 720 min). **A** Colloidal chitin was used as a substrate; **B** NAG_3_ was used as a substrate; **C** NAG_4_ was used as a substrate; **D** NAG_5_ was used as a substrate; **E** NAG_6_ was used as a substrate; **F** NAG_3_ was used as a substrate and reacted using 0.6 μg of purified MtCh509. Lane STD is a standard mixture of NAG_1_–NAG_6_. Lane C is a substrate control without purified MtCh509

A TLC analysis was performed to identify the hydrolysis products produced by MtCh509 (0.3 μg). The reaction mixture was incubated at 55 °C for various times (1–720 min), and hydrolysate spots were observable after 15 min. The color of the spots increased as time passed. Based on the location of the spots in comparison to the NAG_1–6_ standard, the primary component of hydrolysate appeared to be NAG_2_. NAG_1_ was also produced as the incubation continued, and a small quantity of NAG_3_ was produced and then disappeared (Fig. 5A). When chitooligosaccharides were used as the substrate, the major product was also NAG_2_ (Fig. 5B–E). In addition, when more purified MtCh509 (0.6 μg) was used, low amounts of NAG_3_ were converted to NAG_4_ (Fig. 5F). This result was not observed when other chitooligosaccharides were used as the substrate (data not shown).

### HPLC analysis of the hydrolysate produced by MtCh509

**Fig. 6:**
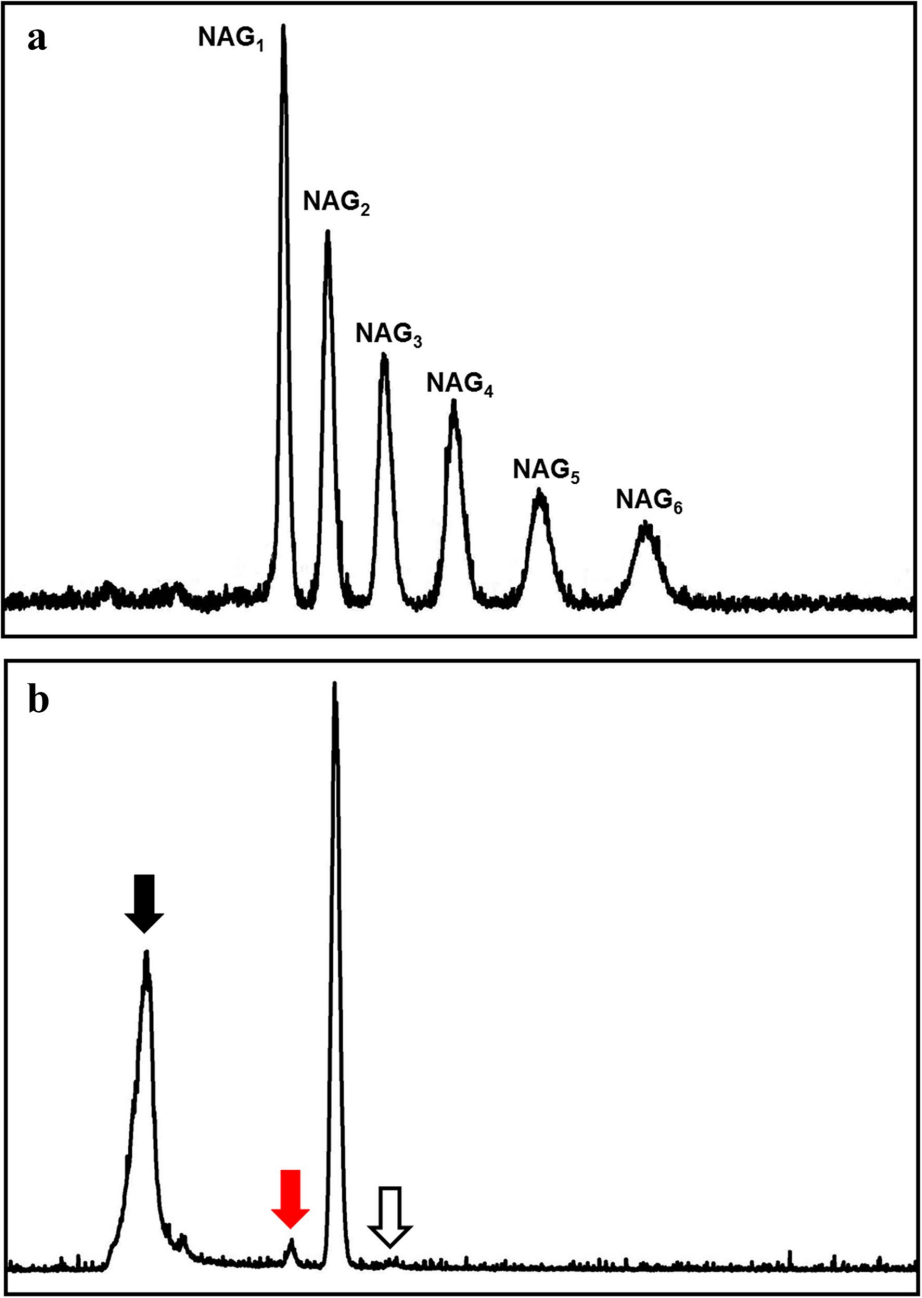
HPLC analysis of the hydrolysate of colloidal chitin generated by purified MtCh509. **a** Standard peaks of NAG_1_–NAG_6_. **b** Hydrolysate of colloidal chitin generated by purified MtCh509 (black arrow, buffer; red arrow, NAG_1_; white arrow, NAG_3_). The reaction mixture (500 μL), which contained 6 μg of purified MtCh509, 50 mM citrate buffer (pH 5.0), and 1% colloidal chitin, was incubated at 55 °C for 12 h

A reaction mixture using colloidal chitin as a substrate was incubated for 12 h at 55 °C and the resulting reaction products were analyzed with a Waters 1500 series HPLC system. The six peaks from left to right are NAG_1_–NAG_6_ (Fig. 6a). The second chromatogram shows the hydrolysate peaks produced from colloidal chitin (Fig. 6b). The peaks marked with black and empty arrows are citrate buffer and NAG_3_, respectively, with the latter peak appearing to be weak. The peak marked with the red arrow is NAG_1_, which is present in small amounts. Because the NAG_2_ peak in the hydrolysate is the highest, the major product of colloidal chitin hydrolysis is NAG_2_.

## Discussion

In this study, we cloned, expressed, and characterized a chitinase from *M. thermotolerans* DAU221, MtCh509. *M. thermotolerans* DAU221 was isolated on the eastern coast of the Republic of Korea [21], and it was subsequently shown to express various proteins, including a carbohydrate esterase [21], a maltotriose-producing α-amylase [32], and an esterase [33]. Recently, the whole genome sequence of DAU221 was completed [34]. In this study, a chitinase from *M. thermotolerans* DAU221 was studied for the first time.

In marine environments, most chitin originates from phytoplankton. Chitin is utilized by native microorganisms as carbon and nitrogen sources and a number of bacteria produce chitinases [35–38]. The products of chitinase-mediated chitin hydrolysis, *N*-acetylglucosamine and chitooligosaccharides, have multiple applications, including in solutions for environmental problems, as antimicrobial or insecticidal agents for the biocontrol of plant pathogens [16], and in physiological processes such as nutritional morphogenesis, pathogenesis [39], parasitism, growth regulation, defense, and immunity [15, 40, 41].

The amino acid sequence of MtCh509 was observed to be similar to that of glycosyl hydrolases from *Simiduia agarivorans* (71%), *Cellvibrio japonicus* (63%), *Cellvibrio mixtus* (60%), *Gilvimarinus agarilyticus* (56%), and *Saccharophagus degradans* (55%) (Fig. 1). The catalytic domain of GH18 enzymes (DXXDXDXE) is conserved across bacteria, fungi, and archaea [42–46], and MtCh509 contains these conserved DXXDXDXE and SXGG motifs (Fig. 1). The consensus sequence of the carbohydrate-binding module (CBM5) is AKWWTK in bacteria [42], and this sequence was observed to be moderately conserved in MtCh509 (A^50^KWWTQ^55^) (Fig. 1). CBM5 can bind substrates through hydrophobic interactions between the aromatic residues of the enzyme and sugar molecules of the substrate [42]. MtCh509 belongs to GH family 18, and it has a catalytic domain containing a TIM-barrel (*β*/*α*)_8_ fold. A comparison of the MtCh509 amino acid sequence to those of SpChiA, SpChiB, and SpChiC from *Serratia proteamaculans* 568 [47] revealed that the *α*/*β* fold insertion in MtCh509 was similar to that in SpChiB. SpChiC lacks the *α*/*β* fold insertion between *β*-sheets 7 and 8 in the TIM-barrel fold. The *α* + *β* insertion leads to a deepening of the substrate-binding groove in the GH family 18 chitinases [11], which may improve enzyme processivity.

MtCh509 was heterologously expressed and purified from *E. coli* BL21 (DE3) without its signal peptide (Fig. 2). Temperature and pH are known to have significant effects on enzyme stability and activity [16]. Typically, chitinases have an optimum temperature of 20–50 °C and are stable up to 55 °C [48, 49]. The purified recombinant MtCh509 showed optimal activity at 55 °C in 50 mM citrate buffer (pH 5.0) (Fig. 3), which is similar to that reported for chitinases from *Massilia timonae* [50], *P. barengoltzii* [49], and *B. licheniformis* SK-1 [51]. Generally, chitinases are stable at up to 50 °C [52–55], and MtCh509 maintained ~ 100% enzymatic activity at 50 °C for up to 2 h. The activity of MtCh509 was slightly lower at 40 °C, and MtCh509 exhibited ~ 80% activity after incubating for 4 h. MtCh509 activity was moderately decreased when incubated for 2 h at 50 °C, and 50% activity was reached before 4 h (Fig. 3b). A chitinase from *Aeromonas veronii*, ChiB565, is also stable at up to 50 °C [56]. Purified MtCh509 showed high activity under sub-acidic conditions, with activity dramatically increased at pH 4.4, and maximal activity was observed at pH 4.6 and 5.0 (Fig. 3c). The optimal pH of MtCh509 is comparable to that of the bacterial chitinases from *M. timonae* (pH 5.0) [50], *Paenibacillus* sp. D1 (pH 5.0) [57], and *Sanguibacter* sp. (pH 4.6) [58]. Purified MtCh509 was stable at sub-acidic pH values and showed moderate to weak activity under neutral and alkaline conditions. The acidic chitinase from *Microbispora* sp. [59] showed similar activities.

Marine microorganisms are tolerant to high salt concentrations, and salt-resistant enzymes are vital to industrial processes that require high-salt conditions [16]. The results of our study showed that MtCh509 is a salt-tolerant chitinase, as it maintained a high level of activity at NaCl concentrations of up to 1 M NaCl. At 1.5 M NaCl, activity decreased to 65%, and at 2 M NaCl, the residual activity was ~ 40%. Then, activity only slightly reduced in 5 M NaCl to 20% (Fig. 4). In comparison, a chitinase from *Halobacterium salinarum* CECT showed maximal activity at 1.5 M NaCl [42].

Metal ions can affect enzyme complex formation, the maintenance/destruction of three-dimensional protein structure, and enzyme stability and activity [60]. Hg^2+^ is known to be a major inhibitor of chitinase activity. It reacts with cysteine residues, specifically in –SH groups, and can change the tertiary structure of a protein [16]. Hg^2+^ strongly reduced the activation of MtCh509, even at low concentrations (Table 1). Many bacterial chitinases, such as those of *Bacillus* sp. DAU101 [6], *Penicillium ochrochloron* MTCC 517 [61], and *Pseudoalteromonas tunicata* CCUG 44952T [62] were strongly inhibited by Hg^2+^. Cu^2+^ catalyzes the formation of intramolecular S–S bridges through auto-oxidation as well as the formation of sulfenic acid [16]. Cu^2+^ (at 10 mM) decreased the activity of purified MtCh509. Similarly, a chitinase from *Chitinibacter* sp. GC72 was highly inhibited by Cu^2+^ [63]. In contrast, another chitinase was observed to be stimulated by Cu^2+^, as the Asp and Glu in the chitinase active site binds 2^+^ ions [64]. According to previous reports, Ca^2+^ generally increases the activity of chitinases from various bacteria, including *Chitinibacter* sp. GC72 [63] and *Bacillus* sp. Hu1 [65]. DTT promotes the destruction of S–S bridges in enzymes, and DTT moderately inhibited MtCh509 activity. This result is comparable to that reported for a chitinase from *Bacillus licheniformis* Mb-2 [66]. EDTA binds to metal ions in solution and acts as a chelating agent, and this compound has been reported to reduce the activity of chitinases produced by *Bacillus* sp. [65] and *Streptomyces* sp. [16].

Organic solvents are divided into two categories, non-polar solvents and polar solvents, which include both polar aprotic solvents and polar protic solvents. In our study, we tested the effects of various organic solvents on the chitinase activity of MtCh509, including acetone, butanol, DMSO, ethanol, isoamyl alcohol, isopropyl alcohol, methanol, hexane, benzene, toluene, and acetonitrile. Some organic solvents (acetone, ethanol, isopropyl alcohol, and acetonitrile) strongly inhibited chitinase activity, whereas others (DMSO, isoamyl alcohol, hexane, benzene, and toluene) enhanced enzyme activity (Table 2). The level of activation for purified MtCh509 was higher than that reported for other bacterial chitinases from *A. hydrophila* SBK1 [41] and *Streptomyces* sp. [16]. Most tested non-polar solvents enhanced the activity of purified MtCh509. These results suggest that hydrophobic interactions contribute to the activation of MtCh509 [16].

To identify the best substrate for MtCh509, the chitinase activity of purified MtCh509 was assayed using different substrates (colloidal chitin, glycol chitin, CMC, and Avicel). The highest activity was observed when glycol chitin was used as a substrate. Compared to glycol chitin, colloidal chitin was hydrolyzed much less (~ 17%). However, the specific activity of purified MtCh509 with colloidal chitin as a substrate was higher than that reported for other chitinases [6, 67]. Very weak activity was observed when CMC was used as a substrate. Similar to ChiB from *Aeromonas veronii* B565, MtCh509 also did not hydrolyze CMC as a substrate [56], and no chitinase activity was detected in the presence of Avicel. Similarly, SmChiA did not bind to Avicel [18].

The *K*_m_ and *V*_max_ values of MtCh509 for colloidal chitin were 9.275 mg/mL and 20.4 U/mg, respectively. The *K*_m_ values of chitinases from other microorganisms were 12.62 mg/mL [68], 47.92 mg/mL for SmChiD of *Serratia marcescens* GPS5 [69], and 35.12 mg/mL for SpChiD of *Serratia proteamaculans* [70]. MtCh509 showed a higher affinity toward colloidal chitin than the other tested substrates. The measured *V*_max_ of MtCh509 is higher than that reported for several bacterial chitinases, including those from *Pseudoalteromonas* sp. DL-6 (13.51 U/mg) [71], *Streptomyces violaceusniger* (6.6 U/mg) [72], and *Bacillus licheniformis* SK-1 (7.03 U/mg) [51].

Hydrolysates were analyzed from the reaction using colloidal chitin and NAG_2_-NAG_6_ chitooligosaccharides as substrates (Fig. 5). When colloidal chitin was used as the substrate (Figs. 5A, 6), NAG_1_-NAG_3_ appeared as products. The major product generated by MtCh509 (0.3 μg) was NAG_2_. Many bacterial chitinases have also produced similar results [1, 38, 54]. MtCh509 did not hydrolyze NAG_2_ (data not shown), suggesting that MtCh509 could not use NAG_2_ as a substrate. Similarly, *Sp* chitinases (*Sp* ChiA, *Sp* ChiB, and *Sp* ChiC) from *Serratia proteamaculans* 568 [47] and FbalChil8A and MvarChi18A from *Ferrimonas balearica* and *Microbulbifer variabilis* [43] also could not use NAG_2_ as a substrate. NAG_3_ (Fig. 5B) was degraded by MtCh509 to NAG_2_ and NAG_1_ within 15 min. NAG_4_ (Fig. 5C) was degraded to NAG_2_, and NAG_5_ (Fig. 5D) was degraded to NAG_3_ and NAG_2_. NAG_6_ (Fig. 5E) was finally degraded to NAG_3_ and NAG_2_ with NAG_4_ observed as an intermediate product. Based on these results, we concluded that MtCh509 from *M. thermotolerans* DAU221 is a processive endochitinase. ChiA from *Bacillus licheniformis* DSM8785 [67] and *Sp* chitinases (*Sp* ChiA, *Sp* ChiB, and *Sp* ChiC) from *Serratia proteamaculans* 568 [47] showed similar hydrolysis patterns. In particular, when the levels of purified MtCh509 were high (0.6 μg), NAG_4_ was synthesized from NAG_3_, as a substrate (Fig. 5F). This result showed MtCh509 has transglycosylation activity. Under other reaction conditions, such as changing substrates, this result was not observed. Many bacterial transglycosylating chitinases have been reported, including *ChiA and ChiB from Serratia marcescens* [73], *Sp*ChiD from *Serratia proteamaculans* [74], *Stm*ChiA from *Stenotrophomonas maltophilia* [18], and *Vh*ChiA from *Vibrio harveyi* [75].

In conclusion, an approximately 60 kDa chitinase from *M. thermotolerans* DAU221 (MtCh509) was heterologously expressed in *E. coli* and purified by His-tag affinity chromatography. The recombinant protein was stable under sub-acidic conditions and at high temperatures and was highly active in the presence of 1 M NaCl, tolerating up to 5 M NaCl. In addition, MtCh509 interacts with several non-polar organic solvents, presumably through hydrophobic interactions. MtCh509 shows specificity for colloidal chitin with crystalline polysaccharides. Based on amino acid sequence and TLC analyses, MtCh509 is an endochitinase. In addition, MtCh509 has transglycosylation activity and produces NAG_4_ from NAG_3_. Thus, MtCh509 can be used for various biotechnological applications.
